# FAVOR-GPT: a generative natural language interface to whole genome variant functional annotations

**DOI:** 10.1093/bioadv/vbae143

**Published:** 2024-09-28

**Authors:** Thomas Cheng Li, Hufeng Zhou, Vineet Verma, Xiangru Tang, Yanjun Shao, Eric Van Buren, Zhiping Weng, Mark Gerstein, Benjamin Neale, Shamil R Sunyaev, Xihong Lin

**Affiliations:** Department of Biostatistics, Harvard T.H. Chan School of Public Health, Boston, MA, 02115, United States; Weston High School, Weston, MA 02493, United States; Department of Biostatistics, Harvard T.H. Chan School of Public Health, Boston, MA, 02115, United States; Department of Biostatistics, Harvard T.H. Chan School of Public Health, Boston, MA, 02115, United States; Department of Computer Science, Yale University, New Haven, CT, 06520, United States; Department of Computer Science, Yale University, New Haven, CT, 06520, United States; Department of Biostatistics, Harvard T.H. Chan School of Public Health, Boston, MA, 02115, United States; Program in Bioinformatics and Integrative Biology, University of Massachusetts Chan Medical School, Worcester, MA, 01605, United States; Program in Computational Biology and Bioinformatics, Yale University, New Haven, CT, 06520, United States; Department of Molecular Biophysics and Biochemistry, Yale University, New Haven, CT, 06520, United States; Analytic and Translational Genetics Unit, Massachusetts General Hospital, Boston, MA, 02114, United States; Program in Medical and Population Genetics, Broad Institute of Harvard and MIT, Cambridge, MA, 02142, United States; Program in Medical and Population Genetics, Broad Institute of Harvard and MIT, Cambridge, MA, 02142, United States; Department of Biomedical Informatics, Harvard Medical School, Boston, MA, 02115, United States; Department of Biostatistics, Harvard T.H. Chan School of Public Health, Boston, MA, 02115, United States; Program in Medical and Population Genetics, Broad Institute of Harvard and MIT, Cambridge, MA, 02142, United States; Department of Statistics, Harvard University, Cambridge, MA, 02138, United States

## Abstract

**Motivation:**

Functional Annotation of genomic Variants Online Resources (FAVOR) offers multi-faceted, whole genome variant functional annotations, which is essential for Whole Genome and Exome Sequencing (WGS/WES) analysis and the functional prioritization of disease-associated variants. A versatile chatbot designed to facilitate informative interpretation and interactive, user-centric summary of the whole genome variant functional annotation data in the FAVOR database is needed.

**Results:**

We have developed FAVOR-GPT, a generative natural language interface powered by integrating large language models (LLMs) and FAVOR. It is developed based on the Retrieval Augmented Generation (RAG) approach, and complements the original FAVOR portal, enhancing usability for users, especially those without specialized expertise. FAVOR-GPT simplifies raw annotations by providing interpretable explanations and result summaries in response to the user’s prompt. It shows high accuracy when cross-referencing with the FAVOR database, underscoring the robustness of the retrieval framework.

**Availability and implementation:**

Researchers can access FAVOR-GPT at FAVOR’s main website (https://favor.genohub.org).

## 1 Introduction

Multi-faceted variant functional annotation plays a pivotal role in the analysis and interpretation of the findings of array-based Genome-Wide Association Studies (GWAS) and WGS studies (Watanabe *et al.* 2017, Li *et al.* 2020, Quick *et al.* 2020). Examples of large scale WGS studies include the Trans-Omics Precision Medicine (TOPMed) Program, UK Biobank, and *All of Us* (Sudlow *et al.* 2015, Karczewski *et al.* 2020, Taliun *et al.* 2021). Variant function annotations can be used for functional fine mapping (Kichaev *et al.* 2014, Schaid *et al.* 2018), partitioned heritability (Finucane *et al.* 2015), polygenic risk scores (PRSs; Marquez-Luna *et al.* 2021), and rare variant association analysis of WGS studies (Li *et al.* 2022).

The Functional Annotation of Variants Online Resources (FAVOR) database and portal (Zhou *et al.* 2023) provides an open access comprehensive online platform for functional annotations of genetic variants, genomic regions and genes across the whole genome. FAVOR efficiently summarizes and visualizes multi-faceted functional annotation data of all possible (approximately nine billion) single nucleotide variants (SNVs), and insertion and deletion variants (Indels) observed in large-scale genome sequencing studies, such as TOPMed, covering the entire human genome. It enables quick and convenient querying at variant, gene, and region levels. FAVOR integrates variant functional information from diverse sources to elucidate the functional attributes of variants, and assists the prioritization of potential causal variants influencing human phenotypes. However, effectively utilizing FAVOR necessitates a certain level of prior specialized knowledge and background. Users are required to possess a fundamental understanding of different annotation metrics and the specific genes or variants they wish to query, in addition to adhering to the correct input formats. Second, there are various terms and scores that users may need to refer to the FAVOR documentation to understand (Zhou *et al.* 2023). Third, the queried results on the FAVOR portal are static with raw annotation results, precluding interactive calculation of summary statistics of interest.

There is a significant need to develop a user-friendly tool to respond to natural language queries, and provide results in an interactive format that are easy to understand without prior knowledge. This will help bridge the gap in accessibility and usability of variant functional annotations in genetics and genomic research. There are increasing interests in leveraging Large Language Models (LLMs; Touvron *et al.* 2023), such as Chat-GPT, GPT-4 (OpenAI 2023) and LLaMA (Touvron *et al.* 2023) in biomedical applications. This transformative technology offers attractive artificial intelligence capabilities. For example, GPT-4 have shown proficiency and intelligence in human interactions, achieved through instruction tuning and feedback-based training. These potentials have ignited significant interest and excitement within the scientific research community toward LLMs (Sallam 2023). In the open-source world, LLaMA has become increasingly popular (Touvron *et al.* 2023). LLaMA3.1’s performance is on par with GPT-4. This advancement shows great potential for researchers seeking to enhance customization. Recently, VarChat (Paoli *et al.* 2024) was introduced to integrate chatbot-based variant search with the publications from PubMed to generate summaries. It is, however, limited to the small subset of variants documented in the published literature. It lacks the ability to query for multi-faceted functional annotations of any variant (SNV) across the human genome, and fails to provide functional information for a large number of variants in WGS studies.

In this paper, we introduce FAVOR-GPT, an interactive tool that leverages knowledge-guided LLMs to enhance the user experience interacting with the FAVOR database. Compared to the competitive products, we selected the ChatGPT API from OpenAI for following reasons. First, it offers high-quality and contextually relevant responses, while boasting rapid response times, ensuring users receive prompt replies to their queries. Second, ChatGPT provides extensive tools available in the JavaScript ecosystem, and its support for function calling makes it an ideal candidate for adopting the Retrieval-Augmented Generation (RAG) approach. It allows to integrate external knowledge sources and our in-house FAVOR APIs seamlessly into the language model’s generation framework, enhancing the accuracy and relevance of the responses. Third, opting for ChatGPT APIs eliminates the need to run a local language model, and reduces the amount of additional responsibilities and complexities, such as hardware requirements, model fine-tuning, and maintenance. ChatGPT offers a more straightforward setup process, enabling us to focus on building our applications rather than managing the underlying infrastructure.

FAVOR-GPT exhibits the ability to understand user inputs in natural language and improve user experience in navigating the FAVOR database and portal. Its inherent flexibility allows it to accommodate a wide range of input formats, ensuring that queries are properly understood. In addition to retrieve query results from the FAVOR database, FAVOR-GPT has several attractive features. When presenting raw annotation results and values, it enriches these findings with relevant background introduction and leverages the natural language explanations generated by LLM (ChatGPT). It also generates summary statistics calculated using the FAVOR database in response to prompts. This integrative approach significantly improves the understanding of functional annotation results, making the utilization of FAVOR easier for researchers. FAVOR-GPT introduces a practical approach to integrating LLMs specifically tailored for variants functional annotation, without the substantial resource requirements of pretraining or fine-tuning large models.

## 2 Methods

FAVOR-GPT was developed with flexibility, resource efficiency, and adaptability in mind. It was made to combine the documentation information with the annotations from the FAVOR database.

**Figure 1.**
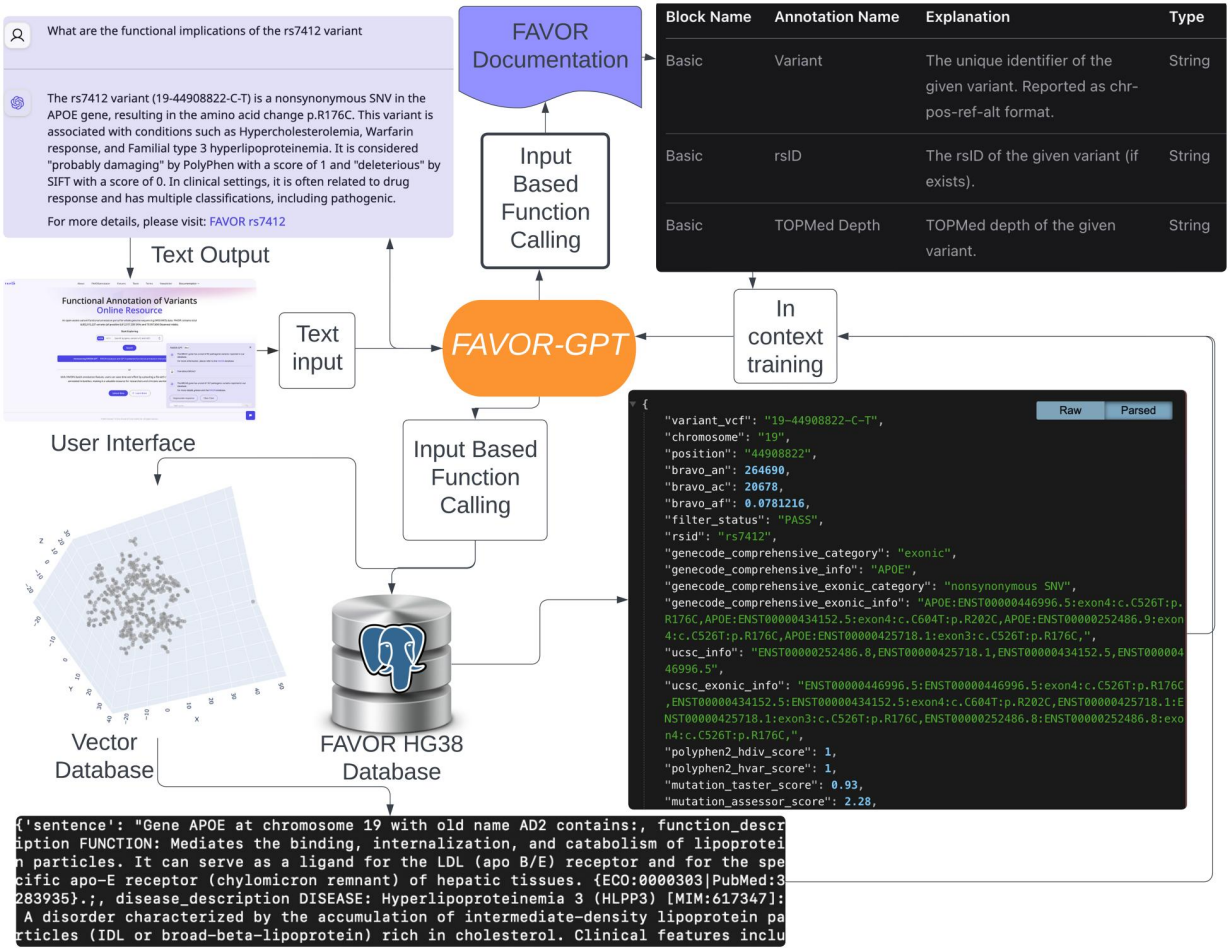
Graphical representation of the FAVOR-GPT workflow. The FAVOR-GPT workflow demonstrates how it converts natural language into structured query syntax and then interprets the query results into clear and fluid natural language.

FAVOR-GPT is implemented based on the Retrieval Augmented Generation (RAG; Guo *et al.* 2023) approach, an AI framework that enhances responses based on an external textual knowledge source. In this case, based on the text query of the user, FAVOR-GPT allows ChatGPT to retrieve textual data in real time from the FAVOR database automatically via the FAVOR API, and thus grounding the LLM on the information from the FAVOR database and documentation and related sources for generating reliable and detailed responses, see Fig. 1. For all gene-related information, FAVOR-GPT utilizes a vector database based on Weaviate to fetch information relevant to the query. Gene information is separated into categories, such as pathway, function, identification, and embedded separately using the “text-embedding-3-small” vectorizer model from OpenAI.

To enhance user comprehension of the annotation results, FAVOR-GPT employs an in-depth analysis of relevant documentation, aligning it with the values obtained from the queries. FAVOR-GPT then employs the ChatGPT APIs to generate natural language explanations of the annotation results from the FAVOR API queries, presenting the information in a format that is easy to understand. Further, FAVOR-GPT can conduct data analysis in response to various queries, such as calculating the number of pathogenic variants in BRCA1. FAVOR-GPT also allows for the user to easily cross-verify the data given in FAVOR-GPT with the database itself. All FAVOR-GPT and FAVOR API documentation can be found at https://docs.genohub.org/.

The workflow of FAVOR-GPT is illustrated in Fig. 1. By harnessing the natural language generation capabilities of Chat-GPT, FAVOR-GPT ensures that users receive not only raw annotation data but also contextual and coherent explanations of multi-faceted functional annotations of variants, genes and genomic regions. The current version of FAVOR-GPT is implemented using the TypeScript programming language (Bierman *et al.* 2014) with the Vercel.AI SDK (Grammel *et al.* 2023), with a deliberate effort to smoothly integrate it into the existing FAVOR user interface which build on the React/Next.js framework. FAVOR-GPT’s presence in the user interface is marked by a clickable floating button placed on the FAVOR website. The source code for the site can be found at https://github.com/zhouhufeng/FAVOR-GPT.

We conducted benchmark testing on FAVOR-GPT by randomly selecting contexts from the vector database and using GPT-4 to create 100 questions based on specific categories like gene function or location. These questions are then answered by both FAVOR-GPT and GPT-4. The answers are assessed based on two metrics: *relevance* and *accuracy*. Relevance measures how well the model’s response addresses the question, with scores of 1 (answer directly pertains to the question), 0.5 (answer tangentially pertains to the question), and 0 (answer does not address the question at all). Accuracy measures how factually correct the answer is, with scores of 1 (completely correct), 0.5 (has mistaken but is largely correct), and 0 (factually incorrect). These scores are determined by a GPT-4 model with access to all the necessary context. To compare the model result, we had a plain GPT-4 model answer the same questions and be evaluated similarly.

## 3 Results

FAVOR-GPT can make any query to satisfy the text inputs. These queries include gene-level functional annotation queries, gene-based variant queries, and variant-specific functional annotation queries. Supplementary Figure S1 shows examples of queries and responses. Users can ask free-form questions like “What is the function of the gene APOE?” and “What is the function of rs942096275?” FAVOR-GPT will provide comprehensive easy-to-understand answers.

FAVOR-GPT is equipped to address computational queries such as analyzing and summarizing data, for instance, gene-level and region-level variant calculations using the FAVOR database. Examples of such computational quires include “What is the range for TP53 gene?,” “How many variants in APOE?,” “How many pathogenic variants in BRCA1?,” “How many loss of function variants in APOE?,” “How many variants in APOE with aPC Epigenetics Repressed > 20?.” These responses are shown in Supplementary Figure S2 and Table S1. These gene-level variant calculations are performed using the TOPMed Bravo variant list, which contains observed variants in TOPMed-BRAVO and is part of the FAVOR database. This is achieved through the FAVOR API, which is designed to handle such specific queries. The FAVOR web interface offers limited gene and region level summary statistics. In contrast, FAVOR-GPT is much more flexible, enabling users to calculate a wide range of customized summary statistics based on their specific queries.

The evaluation of FAVOR-GPT shows good performance in providing variant functional annotation information. FAVOR-GPT had a relevance score of 0.865 and an accuracy score of 0.85, whereas the regular GPT-4 model had a relevance score of 0.5 and an accuracy score of 0.595 (All the examples are placed in Supplementary Table S3). In many cases, the GPT-4 model resorted to saying that it did not know the answer to the question, such as “How many pathogenic variants does BRCA1 have?” which raised the accuracy score to be decently high as “I don’t know.” Although the scores show that FAVOR-GPT still has room for improvement, they also show that the current RAG system by integrating the high quality whole genome variant annotation database FAVOR significantly improves gene-related and variant-related queries and calculations.

## 4 Discussions

We have developed FAVOR-GPT, an interactive interface that leverages Language Model APIs with the multi-faceted variant functional annotation database. It furnishes encompassing annotation results within the FAVOR ecosystem, ensuring that users have access to comprehensive knowledge-guided information and explanations. FAVOR-GPT exhibits relevance and accuracy in interpreting users’ natural language inputs, translating them into structured database queries, and explaining annotation results in natural language and hyperlinks of the sources. Serving as the one of the core interfaces for accessing functional annotation within FAVOR, it is also capable of performing various summary statistics calculations using the data in FAVOR.

The utilization of FAVOR-GPT enables a wider community of researchers to more easily conduct genetics and bioinformatics research. Our efforts to harness the power of Language Model APIs to enhance bioinformatics database usage will be helpful for similar developments in the field. The advent of DNN-driven LLMs represents a valuable force for a new type of interface that improves database user experience. FAVOR-GPT sets an example for navigating large and complex databases of a similar nature. By providing a model for developing and implementing intuitive, natural language-driven interfaces, FAVOR-GPT showcases an effective implementation approach for other specialized knowledge bases to broaden their reach and enhance user experience.

## Supplementary Material

vbae143_Supplementary_Data

## Data Availability

The data and software of FAVOR-GPT underlying this article are available in FAVOR database, at https://favor.genohub.org/ and source code of FAVOR-GPT can be accessed at https://github.com/zhouhufeng/FAVOR-GPT.
