# Supplementary material for: FAVOR-GPT: a generative natural language interface to whole genome variant functional annotations
Source: Bioinform Adv. 2024 Sep 28;4(1):vbae143. doi: 10.1093/bioadv/vbae143 (PMC11461909; doi:10.1093/bioadv/vbae143)
Supplement: vbae143_Supplementary_Data [file vbae143_supplementary_data.docx]

**FAVOR-GPT: A Generative Natural Language Interface to Variant Functional Annotations**

**Supplementary Materials**

Thomas Cheng Li*^1,2, +^*, Hufeng Zhou*^1, +^*, Vineet Verma*^1^*, Xiangru Tang*^3^*, Yanjun Shao*^3^*, Eric Van Buren*^1^*, Zhiping Weng*^4^*, Mark Gerstein*^5,6^*, Benjamin Neale^7,8^, Shamil R Sunyaev*^8,9^*, Xihong Lin*^1,8,10^*

*^1^ Department of Biostatistics, Harvard T.H. Chan School of Public Health, Boston, MA, USA*

*^2^ Weston High School, Weston, MA 02493, USA*

*^3^Department of Computer Science, Yale University, New Haven, CT, USA*

*^4^Program in Bioinformatics and Integrative Biology, University of Massachusetts Chan Medical School, Worcester, MA, USA*

*^5^Program in Computational Biology and Bioinformatics, Yale University, New Haven, CT, USA*

*^6^Department of Molecular Biophysics and Biochemistry, Yale University, New Haven, CT, USA*

*^7^Analytic and Translational Genetics Unit, Massachusetts General Hospital, Boston, MA, USA*

*^8^Program in Medical and Population Genetics, Broad Institute of Harvard and MIT, Cambridge, MA, USA*

*^9^Department of Biomedical Informatics, Harvard Medical School, Boston, MA, USA*

*^10^Department of Statistics, Harvard University, Cambridge, MA, USA*

*^+^ Authors contribute equally to this manuscript.*

**Supplementary Figure 1**. Sample FAVOR-GPT responses. This example shows how FAVOR-GPT effectively interprets user input in natural language, performs precise searches in the FAVOR backend database, and then clearly explains the results in natural language.

**
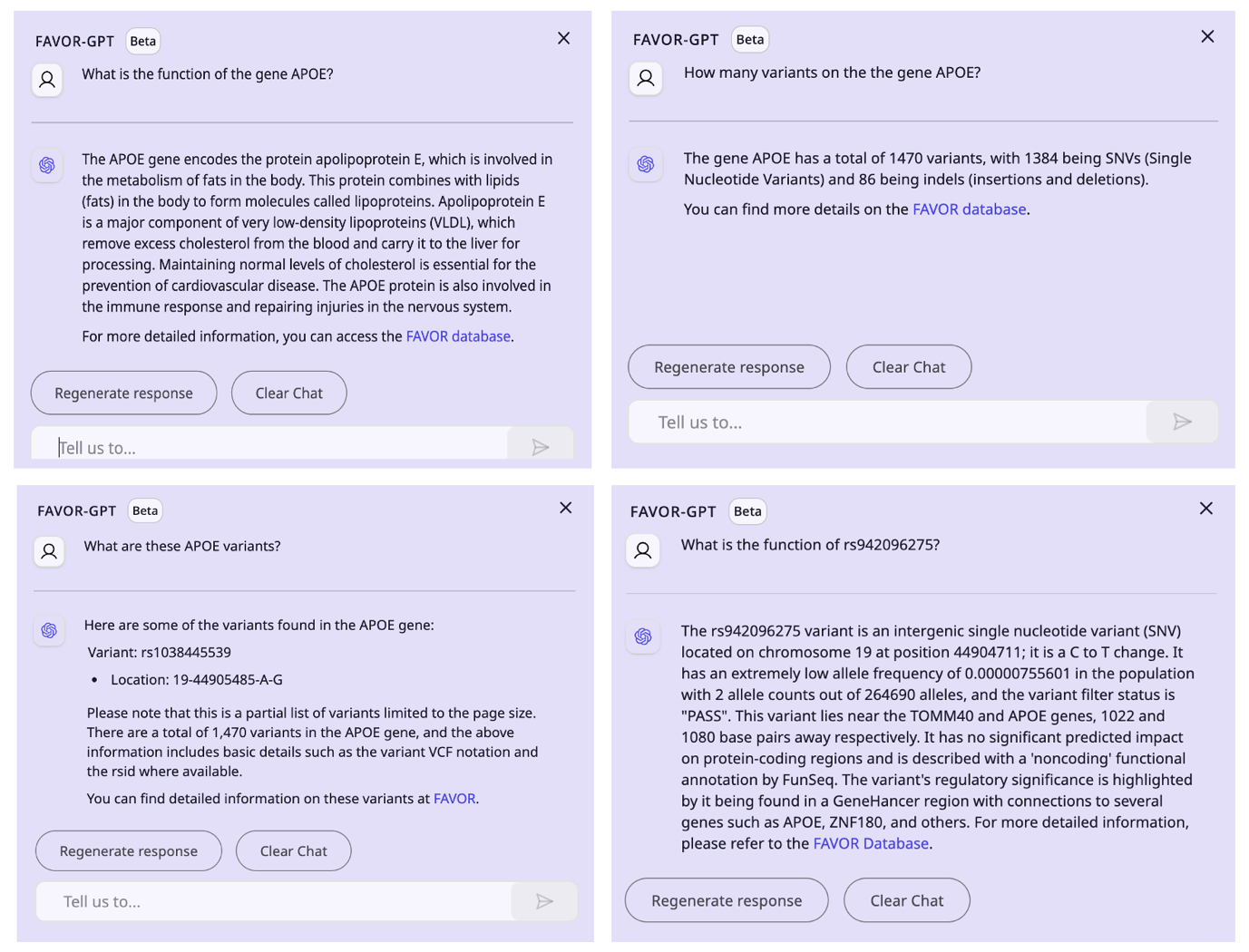
**

**Supplementary Figure 2**. Here are some examples that querying the main web interface cannot provide but are provided by FAVOR-GPT.

*
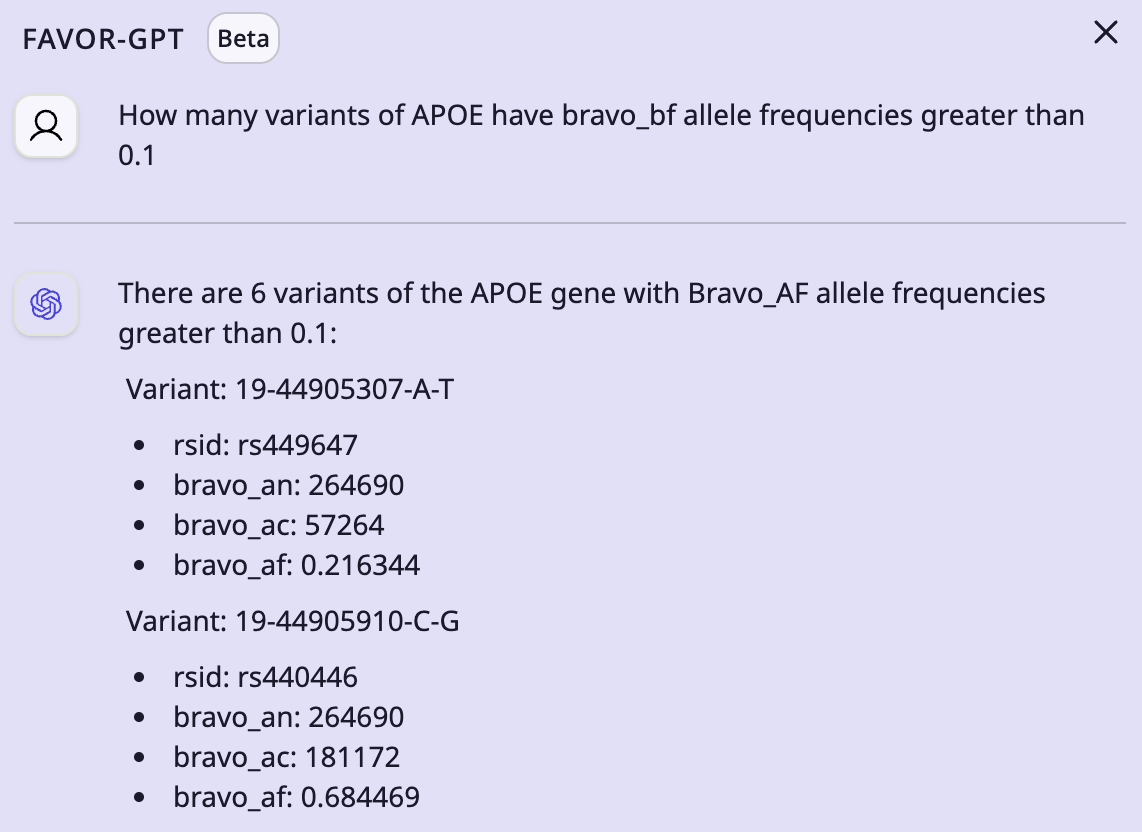
*

*
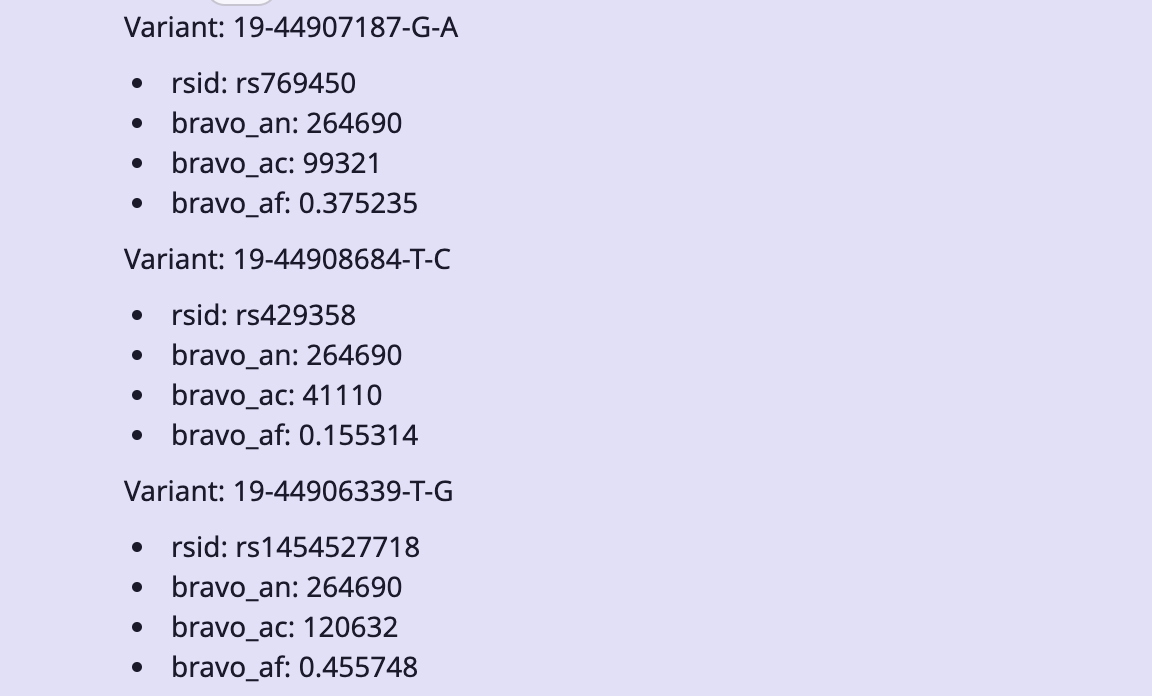
*

*
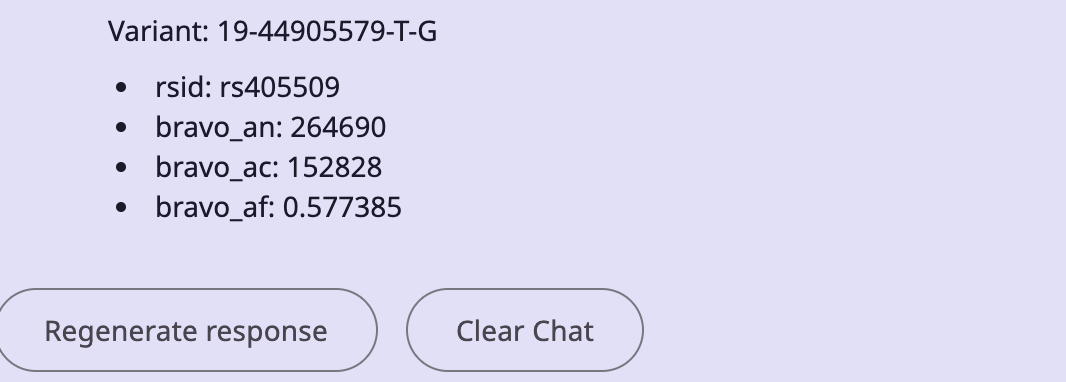
*

*
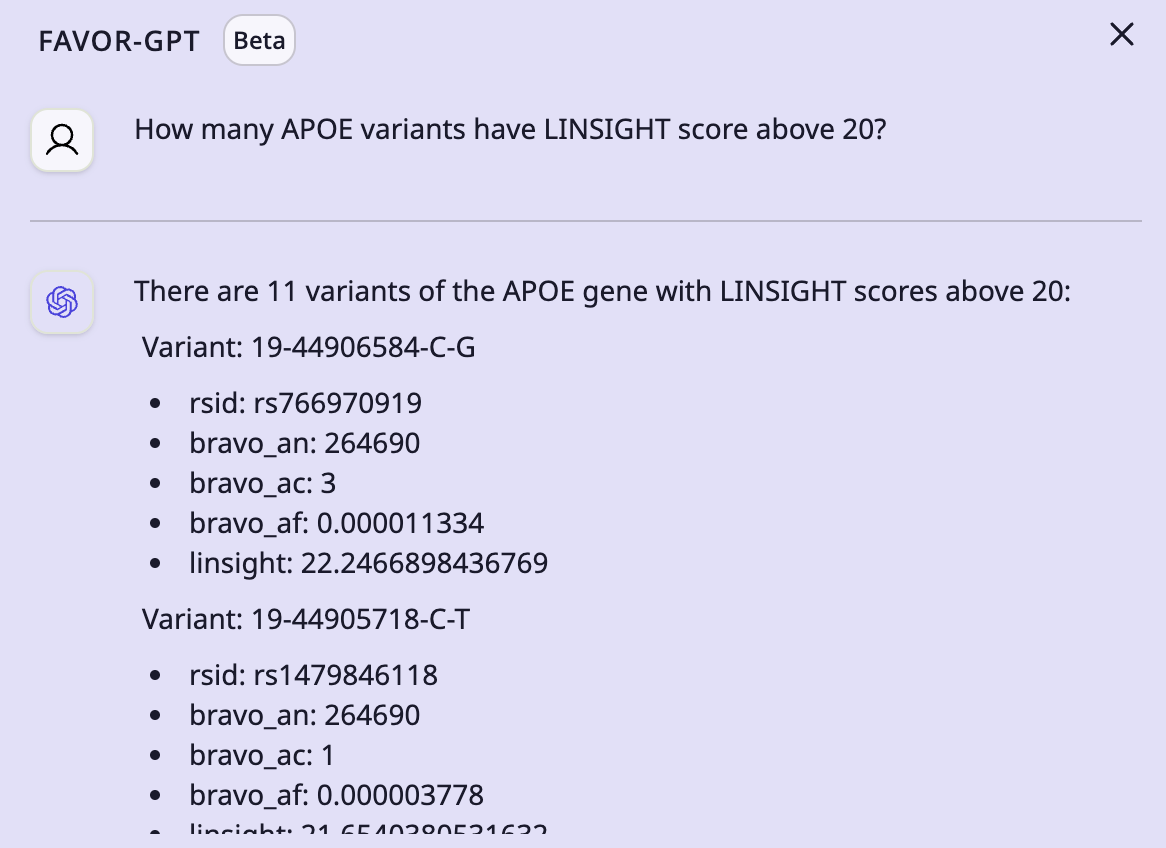
*

*
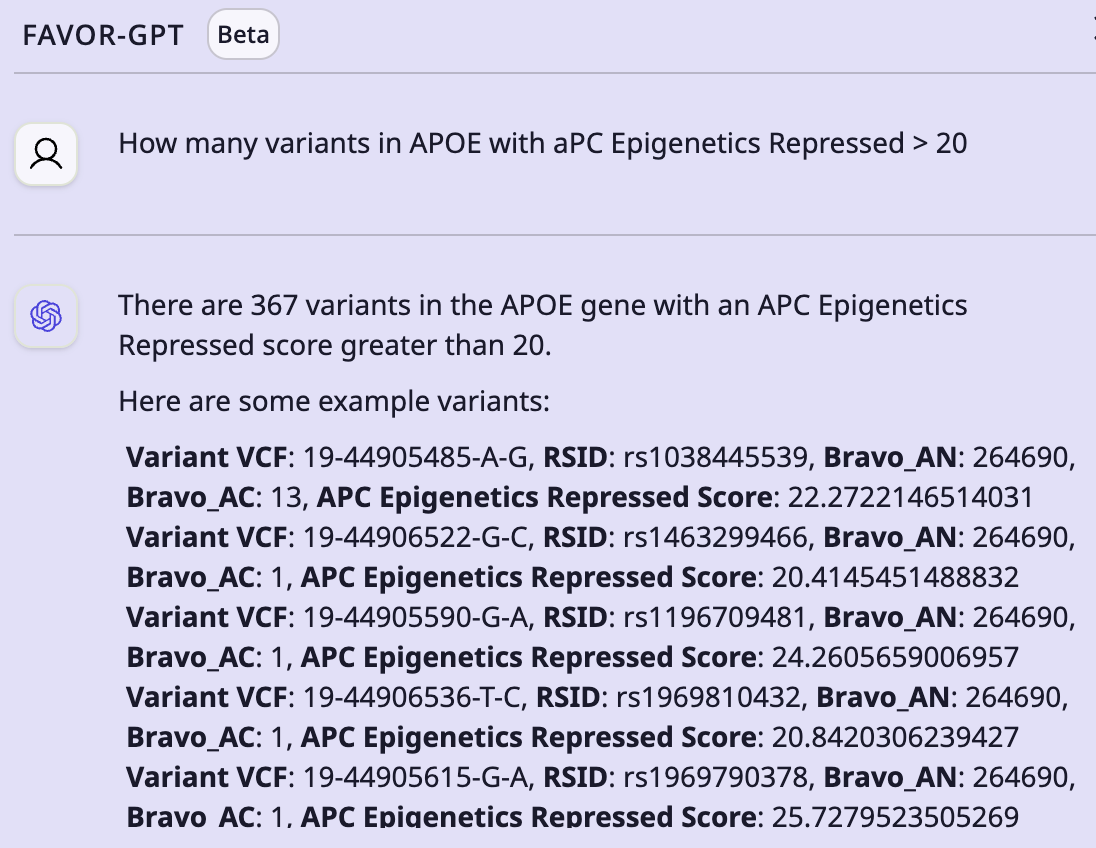
*

***Supplementary* Table 1:** *Sample text responses to three gene-based inputs.*

| Input | Output |
| --- | --- |
| How many variants are in SHOX | The SHOX gene has a total of 238,699 variants. Within these:   - 208,805 are single nucleotide variants (SNVs) - 29,894 are insertions or deletions (indels)   More specific variant details, such as counts of common, low-frequency, and rare variants, are accessible through the FAVOR database. [FAVOR Database SHOX](https://favor.genohub.org/hg38/gene/SHOX/gene-level-annotation/info-and-ids).  (Variant counts derived from Bravo Freeze 8 dataset.) |
| What is the range for TP53 gene? | The TP53 gene is located on chromosome 17 at the cytogenetic band 17p13.1. Its genomic range spans from position 7,668,420 to 7,687,489 on chromosome 17. For additional details, you can refer to the [FAVOR database](https://favor.genohub.org/hg38/gene/TP53/gene-level-annotation/info-and-ids). |
| Give a general description of EGFR gene. | The EGFR (Epidermal Growth Factor Receptor) gene, known by its approved symbol and HGNC ID 3236, is located on chromosome 7 at the chromosomal location of 7p11.2. EGFR plays a critical role in the regulation of cell growth and differentiation. It is implicated in various cancers and is the target of several anticancer therapeutics. The gene spans from genomic position 55,019,016 to 55,211,627 on chromosome 7. The gene has other names, including ERBB1 and ERBB, and is associated with an OMIM ID of 131550. The Uniprot accession number for EGFR is P00533. It is considered an essential gene and is part of the gene family ID 1096 which includes receptor tyrosine kinases. Additionally, EGFR is highly intolerant to loss-of-function mutations, with a pLI score of 0.99968, indicative of its critical function.  For a more in-depth look at the EGFR gene, visit the [FAVOR database](https://favor.genohub.org/hg38/gene/EGFR/gene-level-annotation/info-and-ids). |
| How many pathogenic variants does BRCA 1 have? | The BRCA1 gene has a total of 92 pathogenic variants. You can find more details on the FAVOR database.  (Variant counts derived from Bravo Freeze 8 dataset.) |
| How many pathogenic variants in APOE? | The APOE gene has 11 pathogenic variants in total. Here is the [FAVOR database link](https://favor.genohub.org/hg38/gene/APOE/gene-level-annotation/info-and-ids) for more information.  (Variant counts derived from Bravo Freeze 8 dataset.) |
| How many loss of function variants in APOE? | The APOE gene has a total of 12 observed loss of function (LoF) variants with an expected count of 14.714, and an observed/expected ratio of 0.81553. You can find more detailed information on APOE [here](https://favor.genohub.org/hg38/gene/APOE/gene-level-annotation/info-and-ids).  (Variant counts derived from Bravo Freeze 8 dataset.) |
| How many conserved variants in APOE? | The APOE gene contains a total of 280 conserved variants. You can view more information on these variants in the FAVOR database [here](https://favor.genohub.org/hg38/gene/APOE/gene-level-annotation/info-and-ids).  (Variant counts derived from Bravo Freeze 8 dataset.) |

***Supplementary* Table 2:** *Comparison of the outputs generated by different models (FAVOR-GPT, RAG-T, and RAG-S and using Bravo Freeze 8 variants).*

| Task | Input | Model | Output |
| --- | --- | --- | --- |
| Gene Information | How many pathogenic variants are there in the gene BRCA1? | FAVOR-GPT | The BRCA1 gene has a total of 92 pathogenic variants. For further details, you can visit the FAVOR database [here](https://favor.genohub.org/hg38/gene/BRCA1/gene-level-annotation/info-and-ids). |
|  |  | RAG-T | There are two pathogenic variants. |
|  |  | RAG-S | BRCA1 has two pathogenic variants. |
|  | How many variants are in DMD? | FAVOR-GPT | There are a total of 577,838 variants in the DMD gene, out of which 532,367 are SNVs and 45,471 are indels. You can find more details about these variants on the [FAVOR DMD gene page](https://favor.genohub.org/hg38/gene/DMD/gene-level-annotation/info-and-ids). |
|  |  | RAG-T | There are more than 1000 variants. |
|  |  | RAG-S | More than 1000 |
| Variant Information | What is the pathogenicity of the variant 19-44908822-C-T? | FAVOR-GPT | The variant rs7412 (19-44908822-C-T) is associated with pathogenic outcomes and is clinically significant for drug response, particularly concerning Hypercholesterolemia and Familial type 3 hyperlipoproteinemia, among others. Expert panels in ClinVar have reviewed its significance and computational predictions suggest a deleterious effect on protein function. Furthermore, it influences response to medications such as Warfarin and Atorvastatin. For a comprehensive look at the variant's evidence and classifications, refer to [FAVOR database](https://favor.genohub.org/hg38/variant/19-44908822-C-T/summary/basic). |
|  |  | RAG-T | The variant 19-44908822-C-T may have clinical significance related to a specific health condition. |
|  |  | RAG-S | Pathogenicity for 19-44908822-C-T needs to be evaluated with clinical correlation. |
|  | Give a short description of rs7412 | FAVOR-GPT | Rs7412 is a single nucleotide variant located on chromosome 19 at position 44908822. It is characterized by a cytosine to thymine change (C>T). This variant is found in the APOE gene and is associated with several conditions, including hypercholesterolemia, warfarin response, familial type 3 hyperlipoproteinemia, and atorvastatin response efficacy, among others. Bioinformatic predictions indicate that it is probably damaging with a PolyPhen score of 1 and a deleterious SIFT score. Its clinical significance includes associations with drug response and pathogenicity. The total allele number (AN) reported in the BRAVO database is 264,690, with an allele count (AC) of 20,678, and an allele frequency (AF) of approximately 7.8%.  For more information, you can access the [FAVOR database](https://favor.genohub.org/hg38/rsid/rs7412/summary/basic). |
|  |  | RAG-T | rs7412 is a SNP associated with different forms of ApoE and cholesterol levels. |
|  |  | RAG-S | Variant rs7412 affects the ApoE gene, linked to Alzheimer's risk and lipid profiles |
